# Prolonged inhibition of bladder function is evoked by low‐amplitude electrical stimulation of the saphenous nerve in urethane‐anesthetized rats

**DOI:** 10.14814/phy2.15517

**Published:** 2022-11-21

**Authors:** Zainab Moazzam, Paul B. Yoo

**Affiliations:** ^1^ Institute of Biomedical Engineering (BME) University of Toronto Ontario Canada; ^2^ Department of Electrical and Computer Engineering University of Toronto Ontario Canada

**Keywords:** acetic acid, bladder neuromodulation, electrical stimulation, overactive bladder, overflow incontinence, saphenous nerve

## Abstract

To better understand the effects of saphenous nerve (SN) stimulation on bladder function, we investigated the duration of electrical stimulation as a key variable in eliciting urodynamic changes. SN stimulation is a novel approach to electrically modulating bladder function. In previous animal studies, bladder‐inhibitory responses were evoked by low‐amplitude (25 μA) stimulus pulses applied in short‐duration (10 min) trials and at frequencies between 10 and 20 Hz. Experiments were performed in urethane‐anesthetized rats that were separated into three groups: intravesical saline infusion + SN stimulation (group A), intravesical 0.1% acetic acid infusion + SN stimulation (group B), and intravesical saline infusion + no SN stimulation (group C). Changes in bladder function— basal bladder pressure (*P*_base_), contraction amplitude (Δ*P*), and inter‐contraction interval (*T*_ICI_)—were measured in response to stimulation trials applied for different durations (10, 20, and 40 min). Trials were also repeated at frequencies of 10 and 20 Hz. In group A, longer‐duration (40 min) stimulation trials applied at 10 Hz evoked overflow incontinence (OI) episodes that were characterized by significant changes in *P*_base_ (122.7 ± 9.1%, *p* = 0.026), Δ*P* (−60.8 ± 12.8%, *p* = 0.044), and *T*_ICI_ (−43.2 ± 13.0%, *p* = 0.031). Stimulation‐evoked OI was observed in 5 of 8 animals and lasted for 56.5 ± 10.7 min. In contrast, no significant changes in bladder function were observed in either group B or group C. Our findings show that longer‐duration trials consisting of electrical pulses applied at 10 Hz are important stimulation parameters that elicit inhibitory bladder responses in anesthetized rodents.

## Graphical abstract

Electrical stimulation of the saphenous nerve is a novel approach to modulating bladder function in anesthetized rodents. Previous work showed that low‐amplitude, short duration trials can alter bladder contraction rate and bladder capacity.In this study, we found that increasing the dose of electrical stimulation (extending the duration of stimulation) can further inhibit reflex bladder activity.

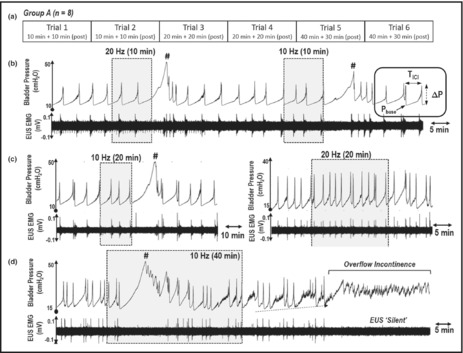

## 1 INTRODUCTION

Neural control of the lower urinary tract (LUT) is achieved via a complex circuitry involving the brain, spinal cord, and the peripheral ganglia (Fowler et al., 2008). These central components control LUT function by establishing efferent and afferent communication via multiple peripheral nerves such as the pelvic, hypogastric, and pudendal nerves (Yoshimura & de Groat, 1997). While the precise therapeutic mechanisms remain unknown, various animal, and clinical studies have shown that electrical stimulation of one or more of these peripheral nerves can effectively modulate bladder function and thereby improve symptoms of LUT dysfunction such as overactive bladder (OAB).

Tibial nerve stimulation is a highly documented electrical neuromodulation modality that has been shown to inhibit bladder activity in healthy anesthetized animals (Kovacevic & Yoo, 2015; Su et al., 2012a) and improve OAB symptoms in patients (Peters et al., 2013; Vandoninck et al., 2003). Studies show that the clinical effects of tibial nerve stimulation are associated with bladder‐inhibitory responses that can be evoked in urethane‐anesthetized animals undergoing isovolumetric (Su et al., 2012b) continuous infusion (Kovacevic & Yoo, 2015), and single urodynamic fills (Choudhary et al., 2016a; Matsuta et al., 2014). Preclinical studies also demonstrate prolonged inhibitory responses, where a decrease or complete inhibition of bladder function persists beyond the period of electrical stimulation (Moazzam et al., 2016; Tai, Shen, et al., 2011).

As an alternative approach, we are investigating a novel nerve target—saphenous nerve (SN)—that when electrically stimulated can elicit bladder inhibitory responses. The SN is a major cutaneous branch of the femoral nerve that provides sensory innervation along the medial surface of the lower leg. In urethane‐anesthetized rats (Franz & Yoo, 2020; Moazzam & Yoo, 2018), we found that short‐duration (10 min), lower‐amplitude (25 μA) stimulation trials can reduce the bladder contraction frequency during continuous bladder infusion; while longer‐duration (30 min), higher‐amplitude (100 μA) stimulation could inhibit reflex bladder contractions (i.e., elicit episodes of overflow incontinence). SN stimulation applied at frequencies between 10 and 20 Hz were most effective in inhibiting bladder function. The inhibitory effects of SN stimulation were also tested in a pilot clinical study involving OAB patients (MacDiarmid et al., 2018), where weekly sessions of percutaneous nerve stimulation resulted in significant improvements in urgency, urge incontinence, and nighttime voiding symptoms.

The aim of this study was to determine the inhibitory effects of using longer‐duration stimulation trials while applying low‐amplitude (25 μA) stimulus pulses. Experiments were performed in anesthetized rats where a suprapubic catheter was used to infuse either saline (healthy model) or acetic acid (hyperactive bladder model; Choudhary et al., 2016b; Chuang et al., 2004; Mitobe et al., 2008) Consistent with our previous work (Kovacevic & Yoo, 2015; Su et al., 2012b), we used a continuous infusion model where stimulation‐evoked changes in urodynamic variables were used to assess bladder function.

## 2 MATERIALS AND METHODS

All experimental protocols were approved by the Animal Use Committee (AUC) at the University of Toronto in accordance with regulations outlined in the Ontario Animal Research Act (Toronto, ON, Canada) and the National Research Council's Guide for the Care and Use of Laboratory Animals.

### 2.1 Experimental set‐up

Acute, non‐survival experiments were performed in 22 adult female Sprague–Dawley rats (250–300 g; Charles River Inc.). Animals were divided as follows: SN stimulation in saline‐infused group (group A; *n* = 8), SN stimulation in a 0.1% acetic acid (AA)‐infused group (group B, *n* = 7), and a control group (group C; *n* = 7). All animals were initially anesthetized under isoflurane (3–5%, O_2_ flow rate: 0.1 ml/min). Following the surgical procedure, the animal was transitioned from isoflurane to urethane (1.2 mg/kg) over a period of 60–90 min (Kovacevic & Yoo, 2015; Maggi & Santicioli, 1986; Moazzam & Yoo, 2018). The core body temperature throughout the experiment was maintained at 37–40°C using a water‐circulating heating pad. Other vitals including the heart rate (300–400 beats/min) and O_2_ level (>97%, 2500 PalmSAT Pulse Oximeter, NONIN Medical) were also monitored. At the end of the experiment, the animal was euthanized with an intra‐cardiac injection of T‐61 (0.3 ml/kg, intra‐cardiac, Merck).

### 2.2 Continuous bladder infusion model

In each experiment, the bladder was surgically exposed following a midline abdominal incision. The bladder dome was catheterized using PE‐50 tubing and secured via a purse‐string suture (6–0 silk sutures). The abdominal incision was then sutured closed in layers using 4–0 sutures. The suprapubic catheter was connected in series with a pressure transducer (Deltran, Model: DPT‐100, Utah Med) and an infusion pump (Harvard Apparatus, Model 70–4500, Pump 11 Elite Infusion). Bladder pressure signals were conditioned using a bridge amplifier (AD Instruments). A pair of de‐insulated stainless steel wire electrodes was inserted in the external urethral meatus (EUS) muscle using a perineal approach (Abud et al., 2015). The EUS electromyogram (EMG) signal was filtered and amplified (Bandwidth: 10–3000 Hz, Gain: 1000) with a low‐noise pre‐amplifier (SRS560, Stanford Research Systems). A subcutaneous needle in the thoracic region served as the electrical ground.

**FIGURE 1:**
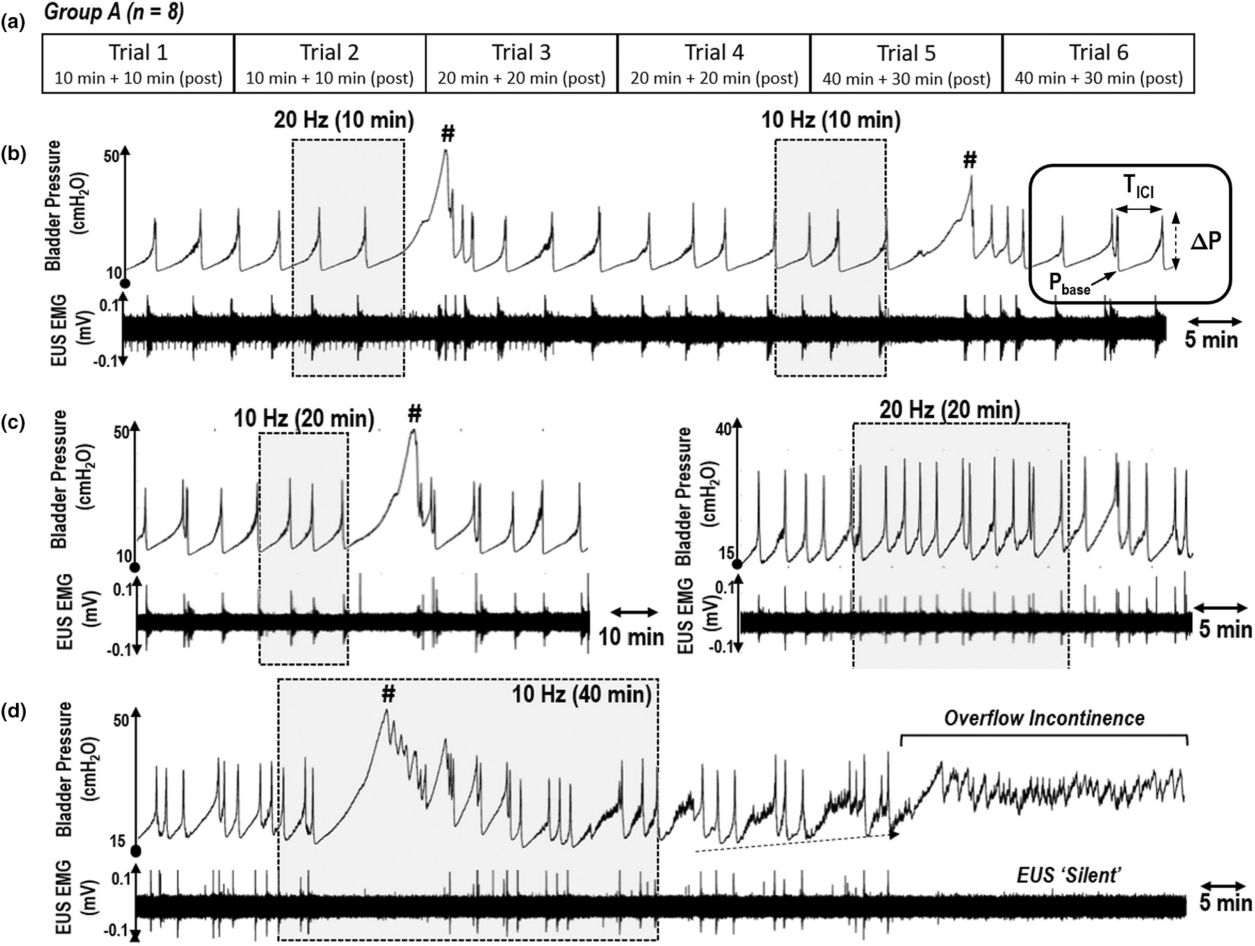
Effects of the duration of SN stimulation on lower urinary tract function. (a) The protocol for group A involved a series of stimulation trials of increasing duration: 10 min ON +10 min OFF, 20 min ON +20 min OFF, and 40 min ON +30 OFF. The stimulation frequency was randomly set at 10 or 20 Hz. Sample data shows that shorter‐duration trials – (b) 10 min and (c) 20 min—elicited extended periods of bladder filling (indicated by **#**). (d) Longer‐duration trials (40 min) resulted in an overflow incontinence (OI) episode during the post‐stimulation period. In this example, a progressive increase in *P*_base_ (dashed arrow) was observed prior to the onset of OI [shaded area = electrical stimulation].

A continuous bladder‐infusion model was used to induce rhythmic reflex bladder contractions by infusing saline via the suprapubic catheter (infusion rate = 0.07 ml/min—0.1 ml/min) (Moazzam & Yoo, 2018). Animals were placed in a supine position. Normal bladder contractions were identified by a simultaneous burst of EUS EMG activity and rapid expulsion of multiple droplets through the urethral meatus. Once the infusion pump was turned on, bladder activity was allowed to stabilize over a period of 1 h. The subsequent 10‐min interval was defined as the baseline period and the following 10‐min period was defined as the pre‐stimulation period. In group B, stimulation trials were conducted while the bladder was constantly infused with 0.1% AA. The effects of SN stimulation were characterized by a loss of bladder function (overflow incontinence, OI) or by changes in urodynamic variables (Figure 1b, rectangular area): (1) the inter‐contraction interval (*T*_ICI_), (2) the basal bladder pressure (*P*_base_), and (3) the contraction amplitude (Δ*P*). All data were acquired digitally (sampling rate = 10 kHz) using PowerLab 16/35 (Model: PL3516, AD Instruments) and analyzed post‐hoc with LabChart (version 7.3.7, AD Instruments) and MATLAB (R2011b, MathWorks Inc.) software.

### 2.3 Surgical instrumentation and stimulation of the SAFN

The SN was accessed through an incision made along the medial aspect of the lower leg. A custom‐fabricated bipolar nerve cuff electrode (inter‐contact distance = 3 mm) was implanted immediately distal to the curvature of the knee. The electrode was connected to an isolated pulse generator (Model 2100, A‐M Systems) to deliver constant‐current pulses. The pulses were monophasic with a pulse width of 200 μs, frequency of 10 or 20 Hz, and an amplitude of 25 μA (Moazzam & Yoo, 2018).

#### 2.3.1 Saline infusion and SN stimulation (group A)

In this experimental group, a total of six SAFN stimulation trials were applied to each of the eight subjects (Figure 1a). Each trial used different combinations of stimulus duration (10, 20, and 40 min) and frequency (10 and 20 Hz). All urodynamic variables were normalized with respect to the pre‐stimulation period.

#### 2.3.2 0.1% AA infusion and SN stimulation (group B)

A total of seven experiments were conducted, where the LUT was chemically irritated with 0.1% AA. In accordance with previous work (Mitsui et al., 2001), relatively low concentration of AA was used in this study to chemically activate afferents in the bladder and urethra during the experiement. In this group, a saline stabilization period (saline infusion: 73.8 ± 3.7 min) was followed by an AA stabilization period (87.7 ± 15.6 min). The subsequent 10‐min of infusion was defined as AA baseline and the next 10 min as the pre‐stimulation period. Unlike group A, only longer‐duration (40 min) SN stimulation trials were applied at 10 and 20 Hz in randomized order (Figure 4a).

#### 2.3.3 Saline infusion and No SN stimulation (group C)

Control experiments were conducted in seven rats where continuous suprapubic infusion of saline was provided but without electrical nerve stimulation. The total duration of the control experiments was consistent with those conducted in the SAFN stimulation group.

### 2.4 Data analysis and significance

All urodynamic parameters were quantified by calculating the respective average value within 10‐min bins. Statistical comparisons of stimulation‐evoked changes in bladder function were made between 10‐min binned data and the pre‐stimulation value. All measurements were normalized with respect to the baseline. In cases where the bladder activity was completely suppressed (i.e., OI), the normalized value for Δ*P* and *T*_ICI_ in their respective bins was assigned a value of zero. The *P*_base_ for that interval was determined by finding the minimum bladder pressure within the corresponding 10‐min bin. We defined an OI episode by (1) absence of reflex bladder contractions, (2) sustained elevation of bladder pressure (passive distension of bladder), (3) absence of EUS EMG bursting activity, and (4) random single droplets of fluid from the urethral meatus (<3 drops in total), all of which lasted for at least 15 min. One‐way ANOVA and paired Student's *t*‐test were used to determine significance (JMP software, SAS Institute Inc.). All data values are presented as mean ± standard error. Significance levels are determined by *p* < 0.05.

## 3 RESULTS

Suprapubic infusion of saline resulted in rhythmic bladder contraction across all experiments and was found to be stable during both the baseline and pre‐stimulation periods. The measured urodynamic variables during baseline (*P*_base_ = 15.3 ± 0.3 cmH_2_O, Δ*P* = 21.7 ± 2.4 cmH_2_O, and *T*_ICI_ = 105.0 ± 15.8 s) were not statistically different from the pre‐stimulation period.

### 3.1 Stimulation‐evoked overflow incontinence

**FIGURE 2:**
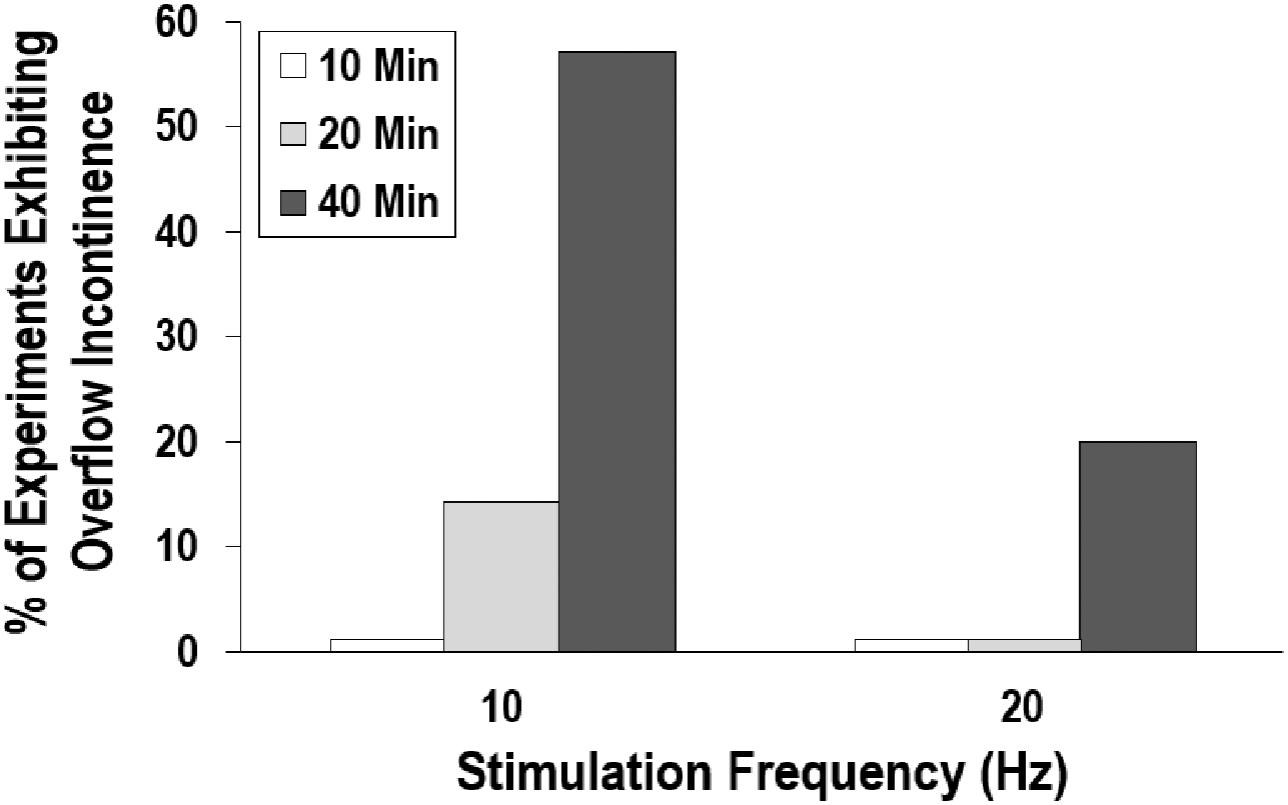
Incidence rate of stimulation‐evoked OI episodes among experiments in group A (*n* = 8). SN stimulation elicited OI most frequently when the stimulation frequency of 10 Hz, where the incidence rate increased with the duration of stimulation: 10 min (0%), 20 min (14%), and 40 min (57%). In contrast, the incidence rate of OI in response to stimulation at 20 Hz was limited to 20%.

Increasing the duration of SN stimulation resulted in OI episodes, which entailed a loss of reflex bladder contractions and bursting EUS EMG activity (Figure 1). OI was elicited in five out of eight animals, where the incidence rate was markedly higher for longer‐duration trials (40 min, Figure 2). SN stimulation applied at 10 Hz (57% of stimulation trials) was also more effective at eliciting OI episodes than stimulation at 20 Hz (20% of stimulation trials). As shown in Figure 1d, OI episodes commonly occurred near the end of the stimulation period and lasted for approximately 56.5 ± 10.7 min (range: 20–89 min). Qualitatively, OI episodes were associated with an increase in *P*_base_ and decreases in both *T*_ICI_ and Δ*P*.

**FIGURE 3:**
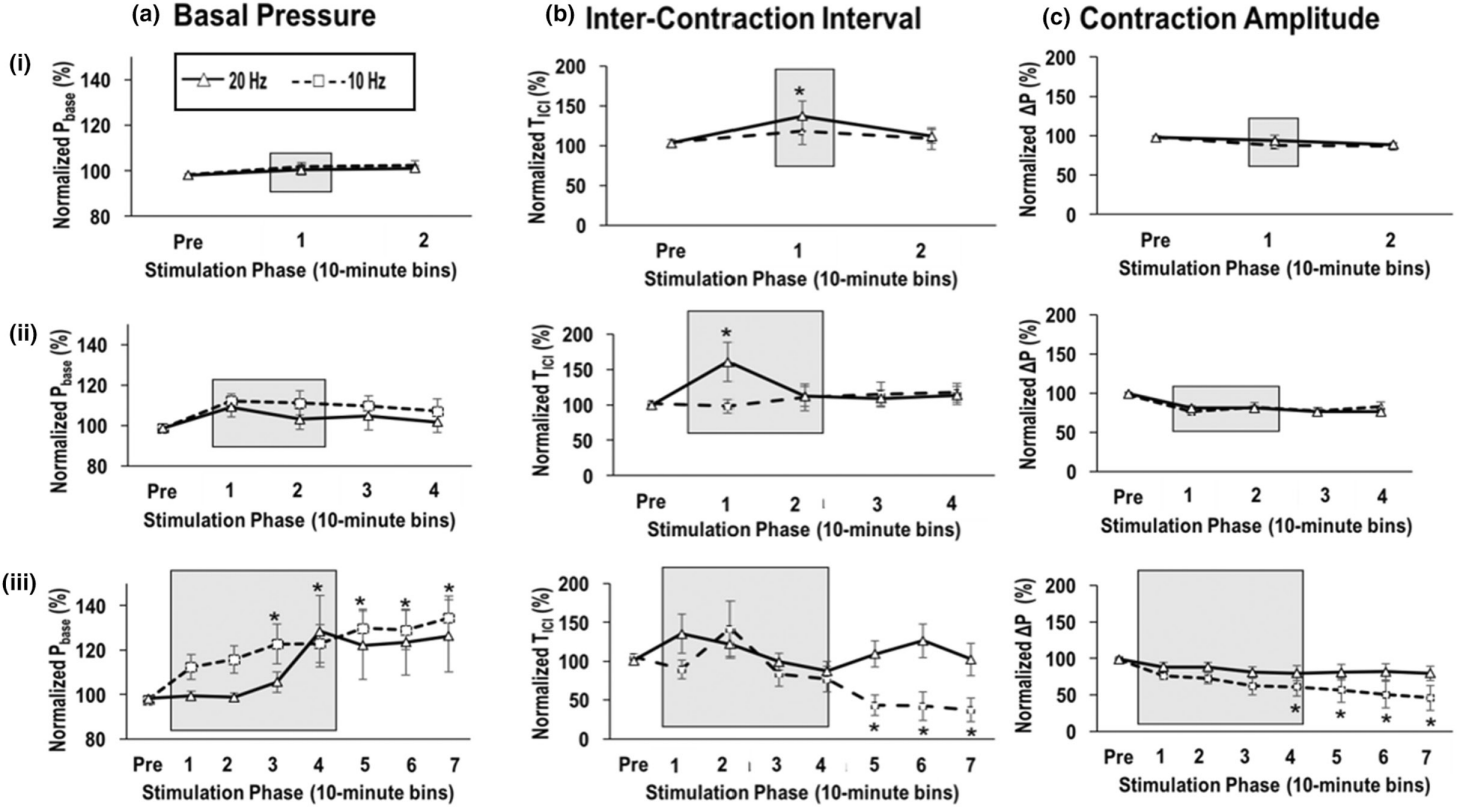
Summary of stimulation‐evoked changes in urodynamic parameters: (column A) basal pressure, (column B) inter‐contraction interval, and (column C) contraction amplitude. Each row of figures corresponds to SN stimulation trials applied for a duration of (i) 10 min, (ii) 20 min, and (iii) 40 min. Data (mean ± SE) were plotted in 10‐min bins corresponding to pre‐stimulation (pre), stimulation (gray area), post‐stimulation periods, and the stimulation frequency were indicated by either a dashed line (10 Hz) or solid line (20 Hz). The effects of short‐duration SN stimulation (20 Hz) were limited to transient increases in *T*_ICI_ during the stimulation period: 10 min trials (137.0 ± 18.7%, *n* = 7, *p* = 0.028) and 20 min trials (160.7 ± 28.1%, *n* = 5, *p* = 0.003). It is noted that short‐duration SN stimulation at 10 Hz had no significant effects (rows i and ii). In contrast, longer‐duration stimulation at 10 Hz elicited significant changes in all three urodynamic parameters (row iii). All parameters were normalized to the saline baseline in each experiment (*p < 0.05, paired Student's *t*‐test).

Changes in urodynamic parameters were affected by OI episodes elicited by SN stimulation at 10 Hz (Figure 3). In contrast to shorter‐duration trials (rows i and ii), the data corresponding to 40 min of SN stimulation (row iii, dashed line) exhibited significant changes in bladder function near the end or after the stimulation period. The *P*_base_ increased significantly by bin #3 (122.7 ± 9.1%, *n* = 6, *p* = 0.03) and remained elevated for at least 30 min after stimulation was stopped (bin 7: 134.4 ± 9.9%, *p* = 0.002). Decreases in the *T*_ICI_ (43.2 ± 13.0%, *n* = 7, *p* = 0.03) and Δ*P* (60.8 ± 12.8%, *n* = 7, *p* = 0.04) also reached significance by bins #5 and #4, respectively. In contrast, SN stimulation applied at 20 Hz had a limited effect on bladder function except for the noted changes in T_ICI_ during shorter‐duration trials (rows i and ii, Figure 3).

### 3.2 Effect of SAFN stimulation in an AA model

**FIGURE 4:**
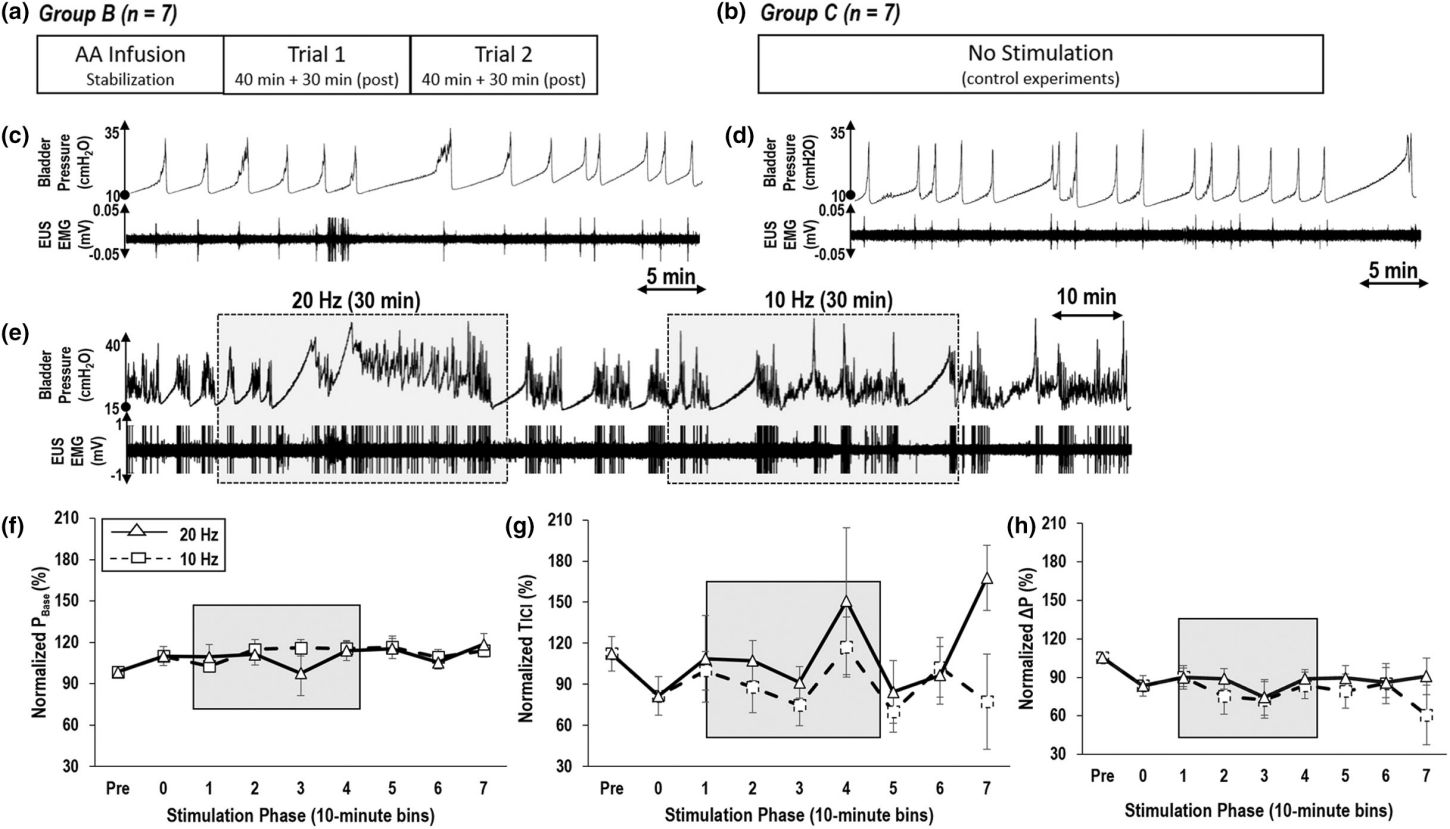
Experimental protocol of (a) SN stimulation in a hyperactive bladder model via suprapubic infusion of 0.1% AA and (b) no stimulation. Control experiments showed no evidence of OI during (c) the initial 40 min and (d) final 40 min of suprapubic saline infusion. SN stimulation had a limited effect on the hyperactive bladder model. (e) In this example, an observable increase in *P*_Base_ occurred during stimulation at 20 Hz but was not considered OI because of bursting EUS EMG activity during bladder pressure spikes. The lack of significant effect of SN in group B animals was quantified by the normalized (f) *P*_base_, (g) *T*_ICI_, and (h) Δ*P*.

Baseline bladder function during infusion of 0.1% AA (average duration of 87.7 ± 15.6 min) resulted in a 30.8 ± 16% decrease in *T*_ICI_, an 11.5 ± 7.3% increase in the *P*_base_, and a 21.9 ± 8.3% reduction in Δ*P* when compared to saline infusion. However, as shown in Figure 4e, longer‐duration SN stimulation failed to elicit any significant changes in urinary function (*P*_base_ and Δ*P*), regardless of the stimulation frequency. There were notable increases in *T*_ICI_ at the end of the stimulation trials (117.2 ± 22.0%, *n* = 7), but the effects were highly inconsistent (Figure 4g).

### 3.3 Control experiments

Continuous suprapubic infusion of saline was performed in seven rats, but without applying SN stimulation. During infusion periods of 6.1 ± 0.2 h (range: 5.5–6.7 h), there were no observed episodes of OI. When comparing the initial and final 40 min of bladder activity in each experiment (Figure 4c,d), there were no statistically non‐significant changes in urodynamic variables: 38.5 ± 19.7% (*p* = 0.283) increase in *T*_ICI_, 1.5 ± 3.0% (*p* = 0.961) increase in *P*_base_, and 2.0 ± 4.3% (*p* = 0.887) increase Δ*P*.

## 4 DISCUSSION

This study extends our knowledge of bladder inhibitory responses that can be elicited by SN stimulation in urethane‐anesthetized rats. By setting a constant stimulation amplitude (25 μA), we found that the duration of stimulation trials can have a significant effect. In group A animals, only longer‐duration stimulation trials were able to evoke OI episodes (incidence rate = 62.5% of experiments), which were urodynamically depicted by a gradual loss of bladder contractility. In contrast, the effects of short‐duration trials were limited to transient increases in *T*_ICI_. Interestingly, higher incidence rates of OI were also associated with stimulation delivered at 10 Hz. These findings were validated by the absence of OI episodes in the control experiments (Group C). In addition, we found that low‐amplitude SAFN stimulation was not effective at inhibiting bladder function in hyperactive bladder model (Group B).

### 4.1 SN‐Mediated inhibitory effects in saline‐infused model

By applying a series of stimulation trials with successively longer‐durations, we were able to characterize the progressively stronger inhibitory effects of SN stimulation. Consistent with our initial studies involving SN stimulation (Moazzam & Yoo, 2018) shorter‐duration trials were able to cause a significant increase in the volume and pressure at which reflex bladder contractions occurred. As illustrated in Figure 2 (indicated by #), the large initial contractions were followed by multiple smaller contractions that emptied the bladder and allowed *P*_Base_ to return to normal levels. With longer‐duration trials, we could observe a gradual change in bladder behavior that resulted in OI (Figure 2c). As depicted in Figure 4 (row iii), bladder voiding became less efficient (increased *P*_Base_ and decreased *T*_ICI_) along with weaker bladder contractility (decreased Δ*P*).

In a continuous bladder filling model, OI is used to characterize a loss of reflex bladder activity such as demonstrated by IV injection of hexamethonium bromide (Maggi & Santicioli, 1986). Amico et al. described this phenomenon as ‘passive urethral dribbling’ which was observed following chronic spinal cord transection at *T*_10_ in adult rats. Durant and Yaksh reported OI as a reversible state of ‘dribbling incontinence’ following intrathecal injection of morphine in un‐anesthetized rats (D'Amico et al., 2011; Yaksh et al., 1986). The latter group concluded that the morphine‐induced atonic phases could be due to the inhibition of primary afferent processing via interaction with the parasympathetic outflow to the bladder leading to detrusor relaxation.

The relatively low‐amplitude (25 μA) stimulus pulses used in this study—which was previously shown to be approximately 1.7 times the SN activation threshold (Moazzam & Yoo, 2018)—suggests that electrical recruitment of low‐threshold (large‐diameter) myelinated fibers is capable of inhibiting urinary function. However, as shown in Figure 1D, the transition from normal reflex bladder contractions to OI was notably relatively slow. By following the *P*_Base_, one can see this parameter gradually increase until reflex activity disappeared. In our previous studies, we typically found the onset of OI to be more abrupt, particularly when higher stimulation amplitudes are used. These include experiments where electrical stimulation was used to recruit small myelinated (e.g., *A*γ) SN fibers (Franz & Yoo, 2020) and unmyelinated tibial nerve C‐fibers (Paquette & Yoo, 2019).

It is interesting that SN stimulation at 10 Hz was effective at evoking OI episodes, given that bladder‐inhibitory responses evoked by other peripheral nerve stimulation targets (i.e. tibial nerve) are also tuned to similar frequencies. For example, Su and colleagues demonstrated suppression of bladder contractions in an isovolumetric bladder model using tibial nerve stimulation at 10 Hz (Su et al., 2012a). Similarly, tibial nerve stimulation trials applied at 10 Hz were found to effectively increase *T*_ICI_ (Kovacevic & Yoo, 2015) and elicit OI episodes (Paquette & Yoo, 2019) using a continuous bladder infusion model in anesthetized rats. Similar bladder‐inhibitory effects by lower frequency tibial nerve stimulation have also been shown in anesthetized cats (Tai, Shen, et al., 2011).

### 4.2 Limited inhibitory effects in the AA‐infusion model

The results of this study showed that low‐amplitude SN stimulation was unable to inhibit hyperactive bladder activity induced by infusion of 0.1% AA. There were no stimulation‐evoked episodes of OI observed in any of the group B experiments. Although it is important to note that intravesical infusion of AA induces OAB‐like symptoms such as an increase in bladder contraction rate (Choudhary et al., 2016a; Su et al., 2013; Tai, Chen, et al., 2011), similarities between the effects of AA and the actual pathophysiology of OAB in patients are unclear. Intravesical infusion of AA functions primarily via activation of the inflammatory receptors within the bladder wall which excites the C‐fibers of the hypogastric nerve (HGN) (Mitsui et al., 2001). Work by Mitsui et al. showed that chemically induced nociception not only activates the spinally mediated HGN circuitry but also exhibits an increase in activity within the periaqueductal gray (PAG) resulting in an increase in the contraction rate (Mitsui et al., 2001, 2003). More importantly, it has been shown that AA induces a localized inflammatory response within the bladder epithelium (e.g., neutrophil infiltration), which at higher concentrations or longer exposure can lead to moderate desquamation and necrosis in the urothelium (Mcmurray et al., 2006; Mitobe et al., 2008). While suitable as a model studying interstitial cystitis (Sudol et al., 2020; Zhao & Nordling, 2004), this irritative bladder model does not provide an accurate approximation of detrusor overactivity associated with OAB (Andersson et al., 2011), and consequently, it is not completely unexpected that low‐amplitude SN stimulation failed to elicit OI in our study. When considering AA‐induced bladder hyperactivity can be inhibited by high‐amplitude peripheral nerve stimulation (Su et al., 2013; Tai, Chen, et al., 2011) or high‐dose oxybutynin (Mitobe et al., 2008b), we predict that repeated high‐amplitude SN stimulation should be able to elicit OI by modulating the efferent parasympathetic drive to the bladder (Franz & Yoo, 2020).

### 4.3 Clinical relevance of anesthetized animal model

Urethane is a suitable anesthetic agent in rodents (Maggi & Meli, 1986) and is considered the gold standard for studying the modulatory effects of peripheral nerve stimulation on urinary function. A dose‐dependent effect of urethane is known, where higher concentrations can cause LUT dysfunction such as loss of bladder contractility and urethral muscle activity. Indeed, Yoshiyama et al showed that higher doses of urethane can cause OI in rats and attributed this bladder modulatory effect in part to glutamatergic mechanisms within the spinobulbospinal reflex pathway (Yoshiyama et al., 2013). Other known mechanisms for eliciting OI in rats include bilateral pelvic nerve transection (Hirotsu et al., 1998) and administration of nicotinic acetylcholine antagonists (Maggi et al., 1986). Although we currently do not understand the precise mechanism of action, preliminary work in our lab has shown that SN stimulation fails to elicit OI in chronic spinal cord transected rats (Gruenspan et al., 2022), suggesting that future work should focus on mechanisms within the brainstem and/or suprapontine structures.

In this study, we verified through control experiments that a continuous bladder infusion model can provide an experimental approach for examining stimulation‐evoked changes in bladder function (Abud et al., 2015; Maggi & Santicioli, 1986; Su et al., 2013). Consistent with previous work in anesthetized rats (Maggi & Meli, 1986), we were able to confirm that the observed changes in bladder function are attributed to electrical nerve stimulation and that anesthesia had minimal effect on eliciting OI. And while potential carry‐over effects were minimized by randomizing the order of stimulation trials with respect to frequency, it is reasonable to expect that the shorter‐duration stimulation trials in group A could have contributed to the effects of longer‐duration trials (refer to Figure 1). Further studies of SN stimulation in OAB patients should help clarify the translatability of our findings.

## 5 CONCLUSIONS

The results of this study showed that electrical stimulation of the SN can induce inhibitory changes in bladder function that depend on the duration of stimulation. Longer‐duration trials applied with a stimulation frequency of 10 Hz were found to be effective at eliciting OI episodes. The low‐amplitude stimulus pulses used to activate the SN in this study were consistent with percutaneous activation of large‐diameter SN fibers in OAB patients (MacDiarmid et al., 2018). We have also shown that these myelinated SN fibers can also be electrically activated by non‐invasive surface electrodes (Sharan et al., 2018). Subsequent preclinical work is needed to better understand the central mechanisms of this SN‐mediated inhibitory reflex.

## FUNDING INFORMATION

Canada Foundation for Innovation (John R Evans Leaders Fund, 31506) and the Government of Ontario (Ontario Graduate Scholarship).

## CONFLICT OF INTEREST

PBY has intellectual property and financial interest related to saphenous nerve stimulation (EBT Medical Inc).

## ETHICS STATEMENT

All animal experiments (protocol 20011674) were approved by the Animal Use Committee of the University of Toronto.

## References

[phy215517-bib-0001] Abud, E. M. , Ichiyama, R. M. , Havton, L. A. , & Chang, H. H. (2015). Spinal stimulation of the upper lumbar spinal cord modulates urethral sphincter activity in rats after spinal cord injury. American Journal of Physiology – Renal Physiology, 308(9), F1032–F1040. 10.1152/ajprenal.00573.2014 25694482 PMC6189748

[phy215517-bib-0002] Andersson, K. , Soler, R. , & Füllhase, C. (2011). Rodent models for urodynamic investigation. Neurourology and Urodynamics, 30(5), 636–646. 21661007 10.1002/nau.21108

[phy215517-bib-0003] Choudhary, M. , van Mastrigt, R. , & van Asselt, E. (2016a). Inhibitory effects of tibial nerve stimulation on bladder neurophysiology in rats. Springerplus, 5(35), 35. 10.1186/s40064-016-1687-6 26835217 PMC4713404

[phy215517-bib-0004] Choudhary, M. , van Mastrigt, R. , & van Asselt, E. (2016b). Effect of tibial nerve stimulation on bladder afferent nerve activity in a rat detrusor overactivity model. International Journal of Urology, 23(3), 253–258. 10.1111/iju.13033 26690557

[phy215517-bib-0005] Chuang, Y. , Yoshimura, N. , Huang, C. , Chiang, P. , & Chancellor, M. (2004). Intravesical botulinum toxin a administration produces analgesia against acetic acid induced bladder pain responses in rats. The Journal of Urology, 172(4), 1529–1532. 10.1097/01.ju.0000137844.77524.97 15371885

[phy215517-bib-0006] D'Amico, S. C. , Schuster, I. P. , & Collins, W. F. (2011). Quantification of external urethral sphincter and bladder activity during micturition in the intact and spinally transected adult rat. Experimental Neurology, 228(1), 59–68. 10.1016/j.expneurol.2010.12.008 21167152

[phy215517-bib-0007] Fowler, C. J. , Griffiths, D. , & De Groat, W. C. (2008). The neural control of micturition. Nature Reviews. Neuroscience, 9(6), 453–466. 10.1038/nrn2401 18490916 PMC2897743

[phy215517-bib-0008] Franz KS , Yoo PB . Transecting the hypogastric nerve to uncover the bladder‐inhibitory pathways involved with saphenous nerve stimulation in anesthetized rats. Autonomic Neuroscience Published online 2020. 10.1016/j.autneu.2020.102672, 226, 102672. 32353706

[phy215517-bib-0009] Gruenspan, G. , Wang, J. , Hacheem, L. , Fehlings, M. , & Yoo, P. (2022). The bladder inhibitory effects of saphenous nerve stimulation are mediated by a supraspinal pathways in anesthetized rodents. In Female Pelvic Medicine & Urogenital Reconstruction (p. BS23). Society of Urodynamics.

[phy215517-bib-0010] Hirotsu, I. , Hayano, C. , & Tani, T. (1998). Effect of muscarinic agonist on overflow incontinence induced by bilateral pelvic nerve transection in rats. Japanese Journal of Pharmacology, 76(1), 109–111. 10.1254/JJP.76.109 9517412

[phy215517-bib-0011] Kovacevic, M. , & Yoo, P. B. (2015). Reflex neuromodulation of bladder function elicited by posterior tibial nerve stimulation in anesthetized rats. American Journal of Physiology‐Renal Physiology, 308(4), F320–F329. 10.1152/ajprenal.00212.2014 25428124

[phy215517-bib-0012] MacDiarmid, S. A. , John, M. S. , & Yoo, P. B. (2018). A pilot feasibility study of treating overactive bladder patients with percutaneous saphenous nerve stimulation. Neurourology and Urodynamics, 37(5), 1815–1820. 10.1002/nau.23531 29464764

[phy215517-bib-0013] Maggi, C. A. , & Meli, A. (1986). The suitability of urethane anesthesia for physiopharmacoligical investigations. Part 3: Other systems and conclusions. Experentia, 42, 531–537. 10.1007/BF01946692 3519271

[phy215517-bib-0014] Maggi, C. A. , & Santicioli, O. M. A. (1986). The nonstop transvesical cystometogram in urethane‐anesthetized rats: A simple procedure for quantitative studies on the various phases of urinary bladder voiding cycle. Journal of Phramacological Methods, 15(2), 157–167. 10.1016/0160-5402(86)90064-1 3702468

[phy215517-bib-0015] Maggi, C. A. , Santicioli, P. , & Meli, A. (1986). The nonstop transvesical cystometrogram in urethane‐anesthetized rats: A simple procedure for quantitative studies on the various phases of urinary bladder voiding cycle. Journal of Pharmacological Methods, 15(2), 157–167. 10.1016/0160-5402(86)90064-1 3702468

[phy215517-bib-0016] Matsuta, Y. , Roppolo, J. R. , de Groat, W. C. , & Tai, C. (2014). Poststimulation inhibition of the micturition reflex induced by tibial nerve stimulation in rats. Physiological Reports, 2(1), e00205. 10.1002/phy2.205 24744884 PMC3967688

[phy215517-bib-0017] Mcmurray, G. , Casey, J. H. , & Naylor, A. M. (2006). Animal models in urological disease and sexual dysfunction animal models of lower urinary tract function and dysfunction. British Journal of Pharmacology, 147, 62–79. 10.1038/sj.bjp.0706630 PMC1751496 16465185

[phy215517-bib-0018] Mitobe, M. , Inoue, H. , Westfall, T. D. , Higashiyama, H. , Mizuyachi, K. , Kushida, H. , & Kinoshita, M. (2008). A new method for producing urinary bladder hyperactivity using a non‐invasive transient intravesical infusion of acetic acid in conscious rats. Journal of Pharmacological and Toxicological Methods, 57(3), 188–193. 10.1016/j.vascn.2007.12.001 18367412

[phy215517-bib-0019] Mitsui, T. , Kakizaki, H. , Matsuura, S. , Ameda, K. , Yoshioka, M. , & Koyanagi, T. (2001). Afferent fibers of the hypogastric nerves are involved in the facilitating effects of chemical bladder irritation in rats. Journal of Neurophysiology, 86(5), 2276–2284. http://www.ncbi.nlm.nih.gov/entrez/query.fcgi?cmd=Retrieve&db=PubMed&dopt=Citation&list_uids=11698518 11698518 10.1152/jn.2001.86.5.2276

[phy215517-bib-0020] Mitsui, T. , Kakizaki, H. , Matsuura, S. , Tanaka, H. , Yoshioka, M. , & Koyanagi, T. (2003). Chemical bladder irritation provokes c‐fos expression in the midbrain periaqueductal gray matter of the rat. Brain Research, 967(1–2), 81–88. 10.1016/S0006-8993(02)04226-9 12650968

[phy215517-bib-0021] Moazzam, Z. , Duke, A. R. , & Yoo, P. B. (2016). Inhibition and excitation of bladder function by Tibial nerve stimulation using a wirelessly powered implant: An acute study in anesthetized cats. The Journal of Urology, 196(3), 926–933. 10.1016/j.juro.2016.04.077 27154823

[phy215517-bib-0022] Moazzam, Z. , & Yoo, P. B. (2018). Frequency‐dependent inhibition of bladder function by saphenous nerve stimulation in anesthetized rats. Neurourology and Urodynamics, 37(2), 592–599. 10.1002/nau.23323 28640440

[phy215517-bib-0023] Paquette, J. P. , & Yoo, P. B. (2019). Recruitment of unmyelinated c‐fibers mediates the bladder‐inhibitory effects of tibial nerve stimulation in a continuous‐fill anesthetized rat model. American Journal of Physiology – Renal Physiology, 317(1), F163–F171. 10.1152/ajprenal.00502.2018 31141398 PMC7139496

[phy215517-bib-0024] Peters, K. M. , Carrico, D. J. , Wooldridge, L. S. , Miller, C. J. , & MacDiarmid, S. A. (2013). Percutaneous tibial nerve stimulation for the long‐term treatment of overactive bladder: 3‐year results of the STEP study. The Journal of Urology, 189(6), 2194–2201. 10.1016/j.juro.2012.11.175 23219541

[phy215517-bib-0025] Sharan, E. , Hunter, K. , Hassouna, M. , & Yoo, P. B. (2018). Characterizing the transcutaneous electrical recruitment of lower leg afferents in healthy adults: Implications for non‐invasive treatment of overactive bladder. BMC Urology, 18(1), 10. 10.1186/s12894-018-0322-y 29439703 PMC5812114

[phy215517-bib-0026] Su, X. , Nickles, A. , & Nelson, D. E. (2012a). Comparison of neural targets for neuromodulation of bladder micturition reflex in the rat. American Journal of Physiology‐Renal Physiology, 303(25), F1196–F1206. 10.1152/ajprenal.00343.2012 22874764

[phy215517-bib-0027] Su, X. , Nickles, A. , & Nelson, D. E. (2012b). Neuromodulation in a rat model of the bladder micturition reflex. American Journal of Physiology – Renal Physiology, 302(4), F477–F486. 10.1152/ajprenal.00515.2011 22049401 PMC4070599

[phy215517-bib-0028] Su, X. , Nickles, A. , & Nelson, D. E. (2013). Neuromodulation attenuates bladder hyperactivity in a rat cystitis model. BMC Urology, 13(70), 1–7. 10.1186/1471-2490-13-70 24314228 PMC4029505

[phy215517-bib-0029] Sudol NT , Guaderrama N , Adams‐Piper E , Whitcomb E , Lane F . Percutaneous tibial nerve stimulation for the treatment of interstitial cystitis/bladder pain syndrome: a pilot study. International Urogynecology Journal Published online 2020. doi:10.1007/s00192-020-04481-4, 32, 2757–2764. 32789810

[phy215517-bib-0030] Tai, C. , Chen, M. , Shen, B. , Wang, J. , Roppolo, J. R. , & De Groat, W. C. (2011). Irritation induced bladder overactivity is suppressed by tibial nerve stimulation in cats. The Journal of Urology, 186(1), 326–330. 10.1016/j.juro.2011.04.023 21600604 PMC3138204

[phy215517-bib-0031] Tai, C. , Shen, B. , Chen, M. , Wang, J. , Roppolo, J. R. R. , & de Groat, W. C. C. (2011). Prolonged poststimulation inhibition of bladder activity induced by tibial nerve stimulation in cats. American Journal of Physiology – Renal Physiology, 300(2), F385–F392. 10.1152/ajprenal.00526.2010 21106856 PMC3044011

[phy215517-bib-0032] Vandoninck, V. , van Balken, M. , Finazzi Agrò, E. , Petta, F. , Micali, F. , Heesakkers, J. P. , Debruyne, F. M. , Kiemeney, L. A. , & Bemelmans, B. L. (2003). Percutaneous tibial nerve stimulation in the treatment of overactive bladder: Urodynamic data. Neurourology and Urodynamics, 22(3), 227–232. 10.1002/nau.10111 12707873

[phy215517-bib-0033] Yaksh, T. L. , Durant, P. A. C. , & Brent, C. R. (1986). Micturition in rats: a chronic model for study of bladder function and effect of anesthetics. AJP Regulatory, Integrative and Compartive Physiology, 251(20), 1177–1185. 10.1152/ajpregu.1986.251.6.R1177 3789199

[phy215517-bib-0034] Yoshimura, N. , & de Groat, W. C. (1997). Neural control of the lower urinary tract. International Journal of Urology, 4(2), 111–125. 10.1111/j.1442-2042.1997.tb00156.x 9179682

[phy215517-bib-0035] Yoshiyama, M. , Roppolo, J. R. , Takeda, M. , & de Groat, W. C. (2013). Effects of urethane on reflex activity of lower urinary tract in decerebrate unanesthetized rats. American Journal of Physiology – Renal Physiology, 304(4), F390–F396. 10.1152/ajprenal.00574.2012 23195677 PMC3566496

[phy215517-bib-0036] Zhao, J. , & Nordling, J. (2004). Posterior tibial nerve stimulation in patients with intractable interstitial cystitis. BJU International, 94(1), 101–104. 10.1111/j.1464-410X.2004.04909.x 15217440

